# High thoracic erector spinae plane block for cervical radicular pain: Does steroid addition enhance outcomes?: A retrospective comparative study

**DOI:** 10.1097/MD.0000000000044749

**Published:** 2025-09-19

**Authors:** Tuba Tanyel-Saraçoğlu, Duygu Karali-Bingül, Burak Erken

**Affiliations:** a Pain Medicine Clinic, Başakşehir Çam and Sakura City Hospital, Istanbul, Turkey; b Department of Anesthesiology and Reanimation, Division of Pain Medicine, University of Health Sciences Başakşehir Çam and Sakura City Hospital, Istanbul, Turkey.

**Keywords:** cervical radiculopathy, chronic pain, nerve block, pain management, steroid, ultrasonography

## Abstract

Cervical radicular pain caused by cervical disc herniation is a common condition that markedly impairs quality of life. This retrospective study aimed to compare the efficacy of high thoracic erector spinae plane block (ESPB) alone versus ESPB combined with steroids in treating unilateral cervical radicular pain caused by cervical disc herniation. Medical records of 89 patients with unilateral cervical radicular pain (C5, C6, or C7 nerve roots) who received ESPB at the T1 level with local anesthetic alone (group L, n = 42) or in combination with steroids (group S, n = 47) were retrospectively analyzed. Pain intensity was measured using the numerical rating scale, and functional impairment was assessed with the neck disability index at baseline and at 4 and 12 weeks post-procedure. Both treatment groups showed significant reductions in numerical rating scale and neck disability index scores compared with baseline (*P* < .001). However, between-group comparisons showed no statistically significant difference at 4th and 12th weeks (*P* > .05). High thoracic ESPB provides effective pain relief and functional improvement in patients with cervical radicular pain, with superior outcomes when steroids are added. This study supports ESPB as a safe alternative treatment for cervical radicular pain, particularly when combined with steroids. Further prospective randomized studies are needed to validate these findings.

## 1. Introduction

Cervical radicular pain, often resulting from nerve root compression due to disc herniation, is characterized by pain radiating from the neck to the corresponding dermatome and may be accompanied by sensory and motor function loss. Disc degeneration and spondylosis can also contribute to compression by narrowing the nerve root exit. The incidence of cervical radicular pain is 85 per 100,000, and it negatively impacts quality of life, leading to productivity loss. The most commonly affected nerve root is C7, followed by C6, with other nerve roots affected less frequently.^[1,2]^

Treatment options include conservative, medical, interventional, and surgical approaches. Cervical epidural steroid injections are effective for patients unresponsive to medical treatment or physical therapy but carry risks of serious complications, such as epidural hematoma, infection, spinal ischemia, and spinal damage.^[3]^

Erector spinae plane blocks (ESPBs), which have been safely and effectively used for acute and chronic pain, were first applied for cervical radicular pain by Restrepo-Garces et al in a pregnant patient, providing effective pain relief over a 12-week follow-up.^[4]^ Hong et al compared the effects of high thoracic ESPBs (T1–T2 level) with a local anesthetic to those of cervical epidural injection with a local anesthetic for pain and disability, observing no significant differences between the groups at the 8-week follow-up.^[5]^

To the best of our knowledge, no studies have examined the combined effects of steroids and ESPBs on pain and functional impairment in cervical radicular pain. Therefore, this study aimed to compare the efficacy of a high thoracic ESPB, using either a local anesthetic alone or in combination with steroids, for pain relief and disability in patients with radicular pain due to cervical disc herniation.

## 2. Methods

### 2.1. Study population

This retrospective cohort study was approved by the Blinded For Review (approval no. KAEK/.16.10.2024.242) and adhered to the principles of the Declaration of Helsinki. The study analyzed the electronic medical records of patients diagnosed with and treated for cervical radicular pain at the hospital’s pain management clinic between January 2023 and October 2024.

#### 2.1.1. Inclusion criteria

- Age between 18 and 65 years.
- Unilateral cervical radicular pain confirmed by magnetic resonance imaging (MRI) and clinical involvement of the C5, C6, or C7 nerve roots due to cervical disc herniation.
- Numeric rating scale (NRS) score > 4 after at least 3 months of conservative treatment.
- Receiving a T1-level ESPB for cervical radicular pain at the pain management clinic.

#### 2.1.2. Exclusion criteria

- Presence of neurological deficits.
- Diagnosis of fibromyalgia syndrome.
- Presence of polyneuropathy, mononeuropathy, or entrapment neuropathy in the affected limb.
- Concomitant cervical spondylosis.
- Coexisting cervical facet joint pain.
- Unilateral cervical radicular pain due to MRI- and clinically confirmed involvement of the C4 or higher nerve roots.
- Joint pathology on the affected side (e.g., shoulder, elbow, or wrist).
- Changes or additions to adjuvant pain management medications within the last 3 months.
- Incomplete 3-month follow-up assessments.
- Requirement of surgical intervention during the follow-up period.
- History of previous cervical surgery.
- Any other interventional treatments within 3 months prior to the procedure.

### 2.2. Outcomes

#### 2.2.1. Primary outcome

##### 2.2.1.1. Numeric rating scale

Pain intensity was assessed using an 11-point NRS, where 0 indicated no pain, and 10 represented the most severe pain. This scale evaluated patients’ subjective pain levels before and after the intervention.

#### 2.2.2. Secondary outcome

##### 2.2.2.1. Neck disability index (NDI)

Functional recovery was measured using the NDI, a validated tool developed by Vernon and Mior and adapted into Turkish by Telci et al^[6,7]^ The NDI consists of 10 sections covering areas such as pain intensity, personal care, lifting, reading, headaches, concentration, work, driving, sleep, and recreational activities. Each section includes 6 items scored from 0 (no disability) to 5 (severe disability). For sections where patients had no relevant experience, they were instructed to leave the items unanswered. The final score was calculated as a percentage of the number of completed questions, reflecting the overall disability level.

### 2.3. Intervention

All interventional procedures included in this retrospective study were performed at our pain clinic by pain medicine specialists with a minimum of 5 years of clinical experience. In accordance with routine physician preferences, procedures in group S were performed by physicians Dr DKB and Dr BE, while those in group L were performed by physician Dr TTS. Written informed consent was routinely obtained from all patients after providing comprehensive information about the procedure. On the day of the procedure, patients were transferred to the operating room, where intravenous access was established, and oxygen saturation, cardiac rhythm, and blood pressure were continuously monitored. Patients were positioned prone, with neck flexion achieved by placing a pillow under the thoracic region to facilitate the cephalad spread of the injection. The caudal-to-cephalad needle insertion approach was used.

Under strict sterile conditions, the transverse process of the first thoracic vertebra was visualized using a convex ultrasound probe (Hitachi, C253 Convex). After administering a local anesthetic, the needle was directed toward the inferior aspect of the T1 transverse process and passed through the trapezius, rhomboid, and erector spinae muscles until it contacted the transverse process. The spread of the injectate was observed between the erector spinae fascia and the transverse process using 2 cc of 0.9% isotonic sodium chloride, with the procedure concluding upon the administration of 20 cc of the solution.

In the local anesthetic alone group, 20 mL of 1% lidocaine was administered, while the steroid combination group received a combination of 16 mL of 1% lidocaine and 4 mL (16 mg) of dexamethasone. After the procedure, all patients were transferred to the observation room and monitored for 2 hours.

### 2.4. Follow-up

Follow-up evaluations were retrospectively reviewed from patient records. NRS and NDI scores, documented in the digital medical records, were assessed at 3 time points: prior to the procedure, and at the 4-week and 12-week follow-up visits. These assessments had been conducted during face-to-face clinical visits as part of routine care.

### 2.5. Statistical analysis

Data analysis was performed using SPSS version 23 (IBM Corp., Armonk, NY). Kolmogorov–Smirnov test was used to assess data normality. Descriptive statistics summarized the study population’s demographic characteristics. Age differences between groups were analyzed using the Mann–Whitney *U* test, while duration of pain was compared using the independent samples *t* test, as it met normality assumptions. Sex distribution was analyzed using the chi-square (*χ*^2^) test. A Mann–Whitney *U* test was used to compare NRS and NDI scores between groups in all time points. Within-group changes in NRS and NDI scores from baseline to posttreatment were analyzed using Friedman and Wilcoxon tests. Statistical significance was set at *P* < .05, and confidence intervals were calculated for all significant findings.

## 3. Results

### 3.1. Study population

The records of 170 patients meeting the inclusion criteria were reviewed. 81 records were excluded for the following reasons: incomplete follow-up (n = 8), spondylosis (n = 13), fibromyalgia (n = 8), polyneuropathy (n = 1), entrapment neuropathy in the affected extremity (n = 1), prior interventional treatment (n = 5), and shoulder joint pathology-related pain on the affected side (n = 3), coexisting cervical facet joint pain (n = 42). Ultimately, 89 patients were included in the analysis, with 47 receiving a block combined with steroids (group S) and 42 receiving a block with only a local anesthetic (group L).

Statistical analyses revealed no significant differences in age, duration of pain and sex differences between the groups (Table 1). The levels of clinically involved and MRI-confirmed symptomatic cervical disc herniation are presented in Table 2.

**Table 1:** Age and sex distribution between groups.

	N (sample size)	Age (mean ± SD)	Female (n, %)	Male (n, %)	Duration of pain (wk)
Group S	47	50.28 ± 5.03	30 (36.2%)	17 (63.8%)	27.23 ± 8.01
Group L	42	49.33 ± 6.3	20 (47.6%)	22 (52.4%)	30.54 ± 7.85
*P* value		.392*	.124†		.053‡

Values are presented as number (%) for categorical variables and as mean ± SD for continuous variables.

Group L = local anesthetic alone group, group S = combination with steroid group.

* *P*-values derived from the Mann–Whitney *U* test represent between-group differences in age (group S vs group L).

† *P*-values from the chi-square test indicate differences in sex distribution between the groups.

‡ *P*-values calculated using the independent samples *t* test assess differences in pain duration between group S and group L.

**Table 2:** Levels of symptomatic cervical disc herniation confirmed by clinical involvement and MRI.

	Group S (n, %)	Group L (n, %)
C5 nerve root	4 (8.5%)	5 (11.9%)
C6 nerve root	21 (44.7%)	17 (40.5%)
C7 nerve root	22 (46.8%)	20 (47.6%)

Values are presented as number (%).

Group L = local anesthetic alone group, group S = combination with steroid group, MRI = magnetic resonance imaging.

### 3.2. Outcomes

#### 3.2.1. Primary outcome

##### 3.2.1.1. Numeric rating scale scores

Within-group comparisons using the Friedman test demonstrated statistically significant changes in both NRS scores over time in both group S and group L (*P* < .001 for each group). Further pairwise analysis with the Wilcoxon signed-rank test revealed significant reductions in NRS scores at both the 4th and 12th weeks compared to baseline in each group (*P* < .001; Table 3).

**Table 3:** Comparison of pre- and post-procedural NRS and NDI scores within groups over time.

Group/measurement	Baseline (mean ± SD)	4th week control (mean ± SD)	12th week control (mean ± SD)	*P*-value*	*P*-value†
NRS					
Group S (n = 64)	7.61 ± 0.7	3.08 ± 1.25	5.65 ± 1.16	<.001	<.001
Group L (n = 67)	7.81 ± 0.63	3.26 ± 1.41	5.69 ± 1.37	<.001	<.001
*P*-value‡	.211	.492	.908		
NDI					
Group S (n = 64)	54.63 ± 8.77	22.29 ± 8.26	36.34 ± 7.88	<.001	<.001
Group L (n = 67)	55.9 ± 9.5	25.38 ± 10.66	39 ± 10.23	<.001	<.001
*P*-value‡	.509	.143	.223		

Values are presented as mean ± SD.

Group L = local anesthetic alone group, group S = combination with steroid group, NDI = neck disability index, NRS = numerical rating scale.

* *P*-values with Wilcoxon-rank signed test represent between pre-procedure and 4th-week results of group S and group L.

† *P*-values with Wilcoxon-rank signed test represent between pre-procedure and 12th-week results of group S and group L.

‡ *P*-values with Mann–Whitney *U* test represent between-group comparisons of NRS and NDI scores at pre-procedure, 4th-week, and 12th-week time points.

In group S, the mean NRS score decreased markedly from 7.61 ± 0.7 at baseline to 3.08 ± 1.25 at 4th week and 5.65 ± 1.16 at 12th week. Similarly, in group L, the mean NRS score declined from 7.81 ± 0.63 at baseline to 3.26 ± 1.41 at 4th week and 5.69 ± 1.37 at 12th week. These findings indicate a sustained and clinically meaningful reduction in pain intensity following the intervention in both groups.

The analysis of NRS scores using the Mann–Whitney *U* test revealed no statistically significant differences between the groups at any of the follow-up time points (baseline *P* = .211; 4th week control *P* = .492; 12th week *P* = .908; Table 3).

##### 3.2.1.2. Neck disability index scores

Within-group comparisons using the Friedman test demonstrated statistically significant changes in NDI scores over time in both group S and group L (*P* < .001 for each group). Subsequent pairwise analyses using the Wilcoxon signed-rank test showed significant improvements in NDI scores at both the 4th and 12th weeks compared to baseline in each group (*P* < .001; Table 3).

In group S, the mean NDI score improved markedly from 54.63 ± 8.77 at baseline to 22.29 ± 8.26 at the 4th week and 36.34 ± 7.88 at the 12th week. Similarly, in group L, the mean NDI score decreased from 55.9 ± 9.5 at baseline to 25.38 ± 10.66 at 4th week and 39 ± 10.23 at 12th week. These findings suggest that both interventions contributed to substantial and sustained improvements in neck-related disability over the follow-up period.

Between-group comparisons indicated no statistically significance between group S and group L at both follow-up intervals (baseline *P* = .509, 4th week *P* = .143; 12th week, *P* = .223; Table 3).

### 3.3. Complications

No adverse events were observed in group L. However, in group S, minimal complications such as transient facial swelling in 1 patient and menstrual irregularity in another patient was reported, both of which resolved without intervention.

## 4. Discussion

Our study demonstrated that both patients receiving a high thoracic ESPB (T1) with or without adjunctive steroids experienced an improvement of NRS and NDI scores for unilateral cervical radicular pain over a 3-month follow-up period. However, between group-comparisons showed no statistically significant difference at any time points. To our knowledge, this study is the first to demonstrate that high thoracic ESPBs, whether combined with steroids or not, significantly improve NRS and NDI scores for cervical radicular pain over a 12-week period.

Previous studies have highlighted the benefits of steroid therapy for cervical radicular pain. A randomized controlled trial comparing oral steroids with placebo showed greater efficacy with oral steroids,^[8]^ while another study demonstrated the superior effectiveness of epidural steroids over intramuscular steroids.^[9]^ Steroids exert anti-inflammatory effects by inhibiting the arachidonic cascade via phospholipase A2, with epidural steroid injections aiming to deliver the medication directly to the target tissue.^[3]^ Also, steroids have been considered effective adjuncts due to their anti-inflammatory properties and their potential to inhibit ectopic discharges from nerve fibers.^[10]^ However, in our study significant clinical additive effect of steroids were not found. Similarly, in the study conducted by Manchikanti et al on patients with cervical radicular pain, epidural injections with local anesthetic, combined with steroids or not were compared over a 12-month follow-up period, and no significant difference in efficacy was found between the 2 groups.^[11]^

A review published in 2022 reported level I evidence supporting that cervical epidural injections, whether local anesthetic combined with steroids or using local anesthetic alone, are effective in providing pain relief and improving functional outcomes.^[12]^ Although cervical epidural injections are recommended treatment for cervical radicular pain, they are associated with serious complications such as epidural hematoma and spinal cord injury.^[13,14]^ Sometimes, loss of resistance may not be achieved due to gaps in the epidural region.^[15]^ Furthermore, unlike ESPB, epidural interventions require discontinuation of anticoagulant and/or antiplatelet therapy prior to the procedure. Moreover, since epidural injections are performed under fluoroscopy guidance, there is an associated radiation exposure for both the patient and practitioner.^[14]^

In a cadaveric study evaluating the drug spread in cervical ESPB, the block performed at the C7 level demonstrated spread to the C5 root.^[16]^ However, computed tomography examination of the ESPB performed at the T2 level for shoulder analgesia revealed extension to even C3 to C4 roots level without phrenic nerve involvement.^[17]^ Based on clinical experience, Diwan et al reported that ESPBs administered at the T1 level reached higher cervical levels than those reported in a cadaveric study, without causing phrenic nerve involvement and could be used safely.^[18]^ In a case series of T2-level ESPBs for chronic shoulder analgesia, computed tomography images revealed contrast agent spread around the C4 to C7 foramina.^[19]^ In our clinical practice, the ESPB is routinely performed at the T1 level, as described by Diwan et al, this level is preferred because it allows for spread to the upper cervical levels while avoiding phrenic nerve involvement, making it a safer option compared to higher cervical injections.^[18]^ We do not perform this block for cervical radicular pain at the C4 level or above.

Local anesthetics at various concentrations have been used—either alone or in combination with particulate or non-particulate steroids—in ESPB applications for chronic pain. To the best of our knowledge, no studies to date have compared different doses of local anesthetics or evaluated the differential effects of particulate versus non-particulate steroids in the treatment of cervical radicular pain. In our clinical practice, 0.25% bupivacaine, either alone or combined with a non-particulate steroid to a total volume of 20 mL, is routinely preferred.^[20]^

The mechanism of action of ESPBs has also been extensively studied. A narrative review in 2021 concluded that the most likely mechanism is the direct effect of the local anesthetic mixture on neural structures through physical spread and diffusion.^[21]^

For the first time, an ESPB was performed at the T3 level in a pregnant patient with cervical radicular pain using 20 mL of a mixture containing 40 mg triamcinolone, 0.5% lidocaine, and 0.25% bupivacaine. This intervention resulted in at least a 50% reduction in pain, which persisted for 12 weeks of follow-up.^[4]^

Hong et al compared cervical epidural injection (CEI) and high thoracic (T2–T3 level) ESPB for cervical radicular pain. Neither group received steroids; both were treated with local anesthetics. The CEI group received 4 mL of 0.1% ropivacaine, and the ESPB group received 20 mL of 0.1% ropivacaine. Over 8 weeks of follow-up, the ESPB demonstrated a therapeutic effect similar to that of the CEI.^[5]^ In our study, this improvement persisted over a longer follow-up period of 12 weeks.

The limitations of our study includes its retrospective design and the absence of a placebo group, which prevents full assessment of the placebo effect in pain reporting. Additionally, the inclusion of patients with subacute or chronic conditions may have introduced variability in their responses to steroid therapy. Moreover, as our study focused exclusively on patients with cervical radicular pain originating from the C5 and lower nerve roots, the findings may not be generalizable to patients with higher cervical levels (C4 and above).

Future studies should aim to conduct larger, prospective and randomized controlled trials with longer follow-up periods, ideally including placebo groups.

Furthermore, investigating the effects of ESPBs at higher cervical levels (C4 and above) could provide valuable insights into their broader applicability for cervical radicular pain.

## 5. Conclusions

In conclusion our study demonstrated that high thoracic (T1 level) ESPB significantly reduced pain intensity and disability in patients with unilateral cervical radicular pain due to cervical disc herniation. Both treatment modalities—ESPB with local anesthetic alone or combined with corticosteroids—proved effective in symptom relief, with no statistically significant difference observed between the 2 groups. To our knowledge, this is the first study to directly compare the efficacy of ESPB with and without steroids for this indication.

These findings highlight ESPB as a promising, minimally invasive therapeutic option for cervical radicular pain. Nonetheless, larger-scale randomized controlled trials with extended follow-up periods are warranted to validate these results and to better define the potential additional effect of corticosteroids in ESPB applications.

## Author contributions

**Conceptualization:** Tuba Tanyel-Saraçoğlu.

**Data curation:** Tuba Tanyel-Saraçoğlu, Duygu Karali-Bingül, Burak Erken.

**Formal analysis:** Tuba Tanyel-Saraçoğlu, Duygu Karali-Bingül.

**Investigation:** Tuba Tanyel-Saraçoğlu, Burak Erken.

**Methodology:** Tuba Tanyel-Saraçoğlu.

**Project administration:** Tuba Tanyel-Saraçoğlu, Duygu Karali-Bingül.

**Resources:** Burak Erken.

**Supervision:** Duygu Karali-Bingül, Burak Erken.

**Writing – original draft:** Tuba Tanyel-Saraçoğlu, Duygu Karali-Bingül.

**Writing – review & editing:** Tuba Tanyel-Saraçoğlu, Duygu Karali-Bingül.

## References

[1] Margetis K Magnus W Mesfin FB. Cervical radiculopathy. In: StatPearls. StatPearls Publishing; 2025. https://www.ncbi.nlm.nih.gov/books/NBK441828/. 28722858

[2] Cohen SP Hooten WM. Advances in the diagnosis and management of neck pain. BMJ. 2017;358:j3221. 28807894 10.1136/bmj.j3221

[3] Peene L Cohen SP Brouwer B. 2. Cervical radicular pain. Pain Pract. 2023;23:800–17. 37272250 10.1111/papr.13252

[4] Restrepo-Garces CE Urrego J Mejia-Loaiza C Giraldo L. The erector spinae plane block for radicular pain during pregnancy. Int J Obstet Anesth. 2019;39:143–4. 30914220 10.1016/j.ijoa.2019.02.009

[5] Hong JH Huh SN. Comparison of pain relief of the cervical radiculopathy between high thoracic erector spinae plane block and cervical epidural injection. Anesth Pain Med (Seoul). 2023;18:406–13. 37919924 10.17085/apm.23064 PMC10635850

[6] Vernon H Mior S. The neck disability index: a study of reliability and validity. J Manipulative Physiol Ther. 1991;14:409–15. 1834753

[7] Telci EA Karaduman A Yakut Y Aras B Simsek IE Yagli N. The cultural adaptation, reliability, and validity of neck disability index in patients with neck pain: a Turkish version study. Spine (Phila Pa 1976). 2009;34:1732–5. 19770615 10.1097/BRS.0b013e3181ac9055

[8] Ghasemi M Masaeli A Rezvani M Shaygannejad V Golabchi K Norouzi R. Oral prednisolone in the treatment of cervical radiculopathy: A randomized placebo controlled trial. J Res Med Sci. 2013;18:S43–6. 23961284 PMC3743318

[9] Stav A Ovadia L Sternberg A Kaadan M Weksler N. Cervical epidural steroid injection for cervicobrachialgia. Acta Anaesthesiol Scand. 1993;37:562–6. 8213020 10.1111/j.1399-6576.1993.tb03765.x

[10] Devor M Govrin-Lippmann R Raber P. Corticosteroids suppress ectopic neural discharge originating in experimental neuromas. Pain. 1985;22:127–37. 4047699 10.1016/0304-3959(85)90173-3

[11] Manchikanti L Cash KA Pampati V Wargo BW Malla Y. Management of chronic cervical disc herniation and radiculitis with fluoroscopic cervical interlaminar epidural injections. Int J Med Sci. 2012;9:424–34. 22859902 10.7150/ijms.4444 PMC3410361

[12] Li B-Z Tang W-H Li Y Zhou L Liu M-G Bao S-X. Clinical efficacy of epidural injections of local anesthetic alone or combined with steroid for neck pain: a systematic review and meta-analysis. Biomed Res Int. 2022;2022:8952220. 35663039 10.1155/2022/8952220 PMC9162875

[13] Li B Tang W Li Y Zhou L Liu M Bao S-X. Clinical efficacy of epidural injections of local anesthetic alone or combined with steroid for neck pain: a systematic review and meta-analysis. Biomed Res Int. 2022;2022:1–13. 10.1155/2022/8952220 PMC9162875 35663039

[14] Manchikanti L Knezevic NN Navani A. Epidural interventions in the management of chronic spinal pain: american society of interventional pain physicians (ASIPP) Comprehensive evidence-based guidelines. Pain Physician. 2021;24:S27–S208. 33492918

[15] Joshi J Roytman M Aiyer R Mauer E Chazen JL. Cervical spine ligamentum flavum gaps: MR characterisation and implications for interlaminar epidural injection therapy. Reg Anesth Pain Med. 2022;47:459–63. 35580934 10.1136/rapm-2022-103552

[16] Elsharkawy H Ince I Hamadnalla H Drake RL Tsui BCH. Cervical erector spinae plane block: a cadaver study. Reg Anesth Pain Med. 2020;45:552–6. 32321860 10.1136/rapm-2019-101154

[17] Diwan S Nair A. Erector spinae plane block for proximal shoulder surgery: a phrenic nerve sparing block! Turk J Anaesthesiol Reanim. 2020;48:331–3. 32864650 10.5152/TJAR.2019.55047 PMC7434336

[18] Diwan S Sala-Blanch X Nair A Shah D. Analyzing cadaveric erector spinae plane block: a compendious approach. Reg Anesth Pain Med. 2021;46:554.1–555. 10.1136/rapm-2020-101881 32972918

[19] Forero M Rajarathinam M Adhikary SD Chin KJ. Erector spinae plane block for the management of chronic shoulder pain: a case report. Can J Anaesth . 2018;65:288–93. 29134518 10.1007/s12630-017-1010-1

[20] Viderman D Sarria-Santamera A. Erector spinae plane block in chronic pain management: a scoping review. Tumori. 2021;107:458–67. 33430714 10.1177/0300891620985935

[21] Chin KJ El-Boghdadly K. Mechanisms of action of the erector spinae plane (ESP) block: a narrative review. Can J Anaesth. 2021;68:387–408. 33403545 10.1007/s12630-020-01875-2

